# p53 Expression, Programmed Death Ligand 1, and Risk Factors in Urinary Tract Small Cell Carcinoma

**DOI:** 10.3389/fonc.2021.651754

**Published:** 2021-04-22

**Authors:** Borivoj Golijanin, Boris Gershman, Andre De Souza, Ohad Kott, Benedito A. Carneiro, Anthony Mega, Dragan J. Golijanin, Ali Amin

**Affiliations:** ^1^Department of Pathology and Laboratory Medicine, The Miriam Hospital, The Warren Alpert Medical School of Brown University, Providence, RI, United States; ^2^Urology Department, Minimally Invasive Urology Institute, The Miriam Hospital, The Warren Alpert Medical School of Brown University, Providence, RI, United States; ^3^Division of Urologic Surgery, Beth Israel Deaconess Medical Center, Boston, MA, United States; ^4^Oncology Department, Lifespan Cancer Institute, The Miriam Hospital, The Warren Alpert Medical School of Brown University, Providence, RI, United States

**Keywords:** bladder, small cell carcinoma, PD-L1, p53, risk factors, survival

## Abstract

**Introduction:** Small cell carcinoma of the urinary tract (SCCUT) is a rare finding with poor clinical course. This study sheds light on the molecular subtype and identifies risk factors in patients diagnosed with SCCUT.

**Methods:** Immunohistochemical expression of immunotherapy target programmed death ligand 1 (PD-L1) and luminal (GATA3), basal (p63), and p53 markers are assessed in patients diagnosed with SCCUT. Univariate analysis identified risk factors. Overall survival (OS) is computed using the Kaplan–Meier method.

**Results:** Tissue was available for 70.2% (33/47). All showed a high PD-L1 expression phenotype. p53 is seen in 93.9% (31/33), mostly as overexpression, GATA3 in 45.5% (15/33), and p63 in 57.6% (19/33). For the entire cohort (*n* = 47), 1-year survival was 59.6%, and the median OS was 17 months. Univariate analysis shows that chemotherapy [hazard ratio (HR) = 0.29, 95% confidence interval (CI) = 0.14–0.61, *p* = 0.001], radical surgery (HR = 0.37, 95% CI = 0.18–0.76, *p* = 0.007), and diagnosis of non-pure SCCUT (HR = 0.44, 95% CI = 0.22–0.86, *p* = 0.02) are favorable prognostic features. Metastasis had negative associations with survival (HR = 2.1, 95% CI = 1.1–4.2, *p* = 0.03).

**Conclusions:** In this series, pure and mixed SCCUT are characterized by p53 overexpression and a high PD-L1 phenotype. Histology of non-pure SCCUT is a positive prognosticator, and radical cystectomy or chemotherapy can improve OS. These findings demonstrate that SCCUT may be eligible for PD-L1 immunotherapy.

## Introduction

Bladder cancer is the fifth most common malignancy, with an estimated incidence of 81,400 new cases in 2020 (1). Recently, advances in the molecular pathogenesis of this disease resulted in effective targeted therapies for advanced bladder cancer. Among these therapies, monoclonal antibodies targeting the transmembrane immune checkpoint protein programmed death ligand 1 (PD-L1) have shown superior overall survival (OS) outcomes when compared with chemotherapy in the second-line setting. Furthermore, ongoing characterization of the molecular and immunohistochemical profile of bladder cancer is leading to newer classification criteria, defining subtypes with predictive and prognostic value.

The focus of this study is on the immunohistochemical subtyping of small cell carcinoma of the urinary tract (SCCUT), a morbid disease with an incidence rate of ~1% of all cases of bladder cancer (2). First described in 1981, our understanding of the etiology and pathophysiology of this rare and aggressive variant has been largely extrapolated from the paradigms for the more common pulmonary small cell carcinoma (2, 3). Like pulmonary small cell carcinoma, SCCUT cells have high rates of proliferation (4). Patients typically present with aggressive tumors, commonly muscle invasive, and there is association with other aggressive variants of urothelial carcinoma (UC) (2, 5). Five-year survival for patients diagnosed with SCCUT is 16–25% (compared to 77% in pure UC) likely due to the metastatic and the heterogeneous nature of this disease (2, 6).

Prior work suggests that UC with a basal gene expression profile translates into poor clinical outcomes. Conversely, the luminal subtype correlates with better OS, whereas the p53 wild-type molecular phenotype is associated with resistance to chemotherapy (7–9). Herein, we identify the immunohistochemical expression of PD-L1, basal-like marker p63, luminal marker of urothelial differentiation GATA3, and tumor suppressor protein p53 and expand on the clinical outcomes in SCCUT.

## Materials and Methods

### Cohort

Patients diagnosed with SCCUT between 1990 and 2019 with at least 1 year of follow-up were included in this institutional review board–approved retrospective study, and clinicopathologic and demographic features were recorded for these patients. All available pathology slides were reviewed by a fellowship-trained urologic pathologist (A.A.) to confirm the diagnosis based on the current World Health Organization classification system and identify a representative block for immunohistochemistry. All cases with any amount of small cell component were included in the study. Clinicopathologic features of patients are detailed in Table 1.

**Table 1:** Clinicopathologic features of all patients diagnosed with SCCUT (*n* = 47) and of patients with tissue available (*n* = 33).

		**All patients**	**Patients with tissue available**	**ANOVA *p* value**
No. of patients		47	33	—
Survival (months)	Median	16	17	0.77
	Mean	26.77	28.64	
	SD	27.4	28.9	
	IQR	6, 46	9, 46.5	
Age (years)	Median	72	73	0.99
	Mean	72.19	72.15	
	SD	10.43	9.2	
	IQR	63, 80	63.5, 80	
Sex	Female	14 (29.8%)	10 (30.3%)	0.96
	Male	33 (70.2%)	23 (69.7%)	
Chemotherapy	None	10 (21.3%)	7 (21.2)	0.39
	Etoposide and carboplatin	18 (38.3%)	13 (39.4%)	
	MVAC	7 (14.9%)	3 (9.1%)	
	Etoposide, carboplatin, and cisplatin	1 (2.1%)	1 (3%)	
	Gemcitabine and cisplatin	5 (10.6%)	4 (12.1%)	
	Carboplatin and paclitaxel	2 (4.3%)	2 (6.1%)	
	Etoposide and cisplatin	3 (6.4%)	3 (9.1%)	
	Gemcitabine only	1 (2.1%)	—	
Surgery	TUR only	30 (63.9%)	19 (57.6%)	0.62
	RC	16 (35%)	13 (39.4%)	
	Nephro-U	1 (2.1%)	1 (3%)	
Histology	Pure SCCUT	18 (38.3%)	14 (42.4%)	0.72
	Mixed	29 (61.7%)	19 (57.6%)	
Primary site	Bladder	46 (97.8%)	32 (97%)	0.81
	Ureter	1 (2.1%)	1 (3%)	
Recurrence	No recurrence	27 (57.4%)	15 (45.5%)	0.3
	History of recurrence	20 (42.6%)	18 (54%)	
Pathologic stage	Tx	4 (8.5%)	4 (12.1%)	0.78
	T1	3 (6.4%)	2 (6.1%)	
	T2	17 (36.2%)	7 (21.2%)	
	T3	17 (36.1%)	15 (45.5%)	
	T4	6 (12.8%)	5 (15.2%)	
Nodes^*^	N0	30 (63.9%)	21 (63.6%)	0.74
	N1	11 (23.4%)	9 (27.3%)	
	N2	5 (10.6%)	3 (9.1%)	
	N3	1 (2.1%)	—	
Metastasis	M0	39 (83%)	29 (87.9%)	0.37
	M1	8 (17%)	4 (12.1%)	

*Mean, standard deviation, median, IQR, frequency, and percentages are listed where applicable. No significant differences exist between the expanded cohort and the patients with available tissue. Pathological TNM staging information is based on the guidelines of the AJCC 7th edition Cancer Staging Manual. p ≤ 0.05 is considered significant*.

* *The one upper tract SSCUT is an N2*.

*MVAC, methotrexate, vinblastine, adriamycin and cisplatin; TUR, transurethral resection; RC, radical cystectomy; SCCUT, small cell carcinoma of the urinary tract; Nephro-U, nephroureterectomy*.

### Immunohistochemistry

4-μm-thick sections were cut and mounted onto positively charged glass slides (Thermo Fisher Scientific, Waltham, MA, USA). Slides were baked in an Isotemp (Thermo Fisher Scientific) oven overnight at 37°C. The next day, slides were loaded onto the Ventana Discovery XT (Ventana Medical Systems Inc., Tucson, AZ, USA) for detection of the antibody using the Discovery IHC DABMap kit (Ventana Medical Systems Inc.).

Antigen retrieval was performed using standard cell conditioning 1 (Tris/borate/EDTA), including head induced epitope retrieval in Tris-EDTA buffer pH 7.8 at 95°C for 44 min. Slides were incubated with Endogenous Blocking Kit (Ventana Medical Systems Inc.) for 10 min. Slides were removed from the Ventana Discovery XT and put into soap and DI mixture followed by a 5-min wash using DI. Washed slides were put in hematoxylin for 30 s, followed by DI for 5 min, 0.2% ammonia water for 1 min, and then washed with DI for an additional 5 min. Washed slides were dehydrated and cover slipped.

For PD-L1 expression, the primary antibody used was anti-PD-L1 (Abcam, ab174838, Cambridge, MA, USA). A dilution of 1:1,500 was manually titrated onto the slides and incubated for 30 min. The universal secondary antibody was automatically dispensed and incubated for 30 min. Staining on tumor cells (TCs) and infiltrating immune cells (ICs+) was evaluated for percent of cells expressing PD-L1. Lymph node and tonsil tissue were used as positive controls.

Interpretation of positive PD-L1 staining was performed based on vendor recommendations. In brief, PD-L1 status was determined by the percentage of TCs with any membrane staining above background or by the percentage ICs+ at any intensity above background. The percent of tumor area occupied by any tumor-associated ICs [ICs present (ICPs)] was used to determine ICs+, which is the percent area of ICPs exhibiting PD-L1–positive IC staining. PD-L1 status was considered high if any of the following were met: (1) ≥25% of tumor cells exhibit membrane staining; (2) ICPs >1% and ICs+ ≥25%; or (3) ICPs = 1% and ICs+ = 100%.

To categorize the tissue based on molecular subtype, the GATA3 antibody (L50-823, Biocare Medical, Pacheco, CA, USA) was used to identify luminal subtype, the p63 antibody (4A4, Biocare Medical) applied to classify the specimens as basal, and the p53 antibody (DO-7, Dako, Santa Clara, CA, USA) was employed to evaluate p53 expression. Expression of nuclear p63 and GATA3 was assessed using the three-point immunoreactive scoring system looking for the extent (0 = none, 1 = <10% of cells, 2 = 10–50%, and 3 = >50%) and strength (0 = negative, 1 = weak intensity, 2 = intermediate intensity, 3 = strong intensity) in each specimen. Staining of p53 nuclear protein expression was ranked using a three-point system (0 = loss of expression, 1 = wild-type expression, and 2 = overexpression).

### Statistical Analysis

Clinicopathologic and demographic features were summarized using frequency counts and percentages. These variables were correlated with expression of the aforementioned immunohistochemical stains. OS was estimated using the Kaplan–Meier method. Cox proportional hazards models were used to evaluate the correlation of clinicopathologic features and immunohistochemical markers on survival. A univariate Cox regression model was performed on all covariates. Statistical analyses were performed using RStudio 1.3.1073 (RStudio, Boston, MA, USA). *p* < 0.05 was considered significant.

## Results

Two cohorts were generated: the major cohort includes all patients diagnosed with SCCUT (*n* = 47), and the minor cohort includes patients with tissue available (*n* = 33).

### All Patients

A total of 47 patients diagnosed with SCCUT were identified between 1990 and 2019 (Table 1). Median age at diagnosis was 72 years [interquartile range (IQR) = 63–80 years]; 70.2% (33/47) were male; median OS was 16 months [IQR = 6–46 months, 95% confidence interval (CI) = 12–28 months]; and median reverse Kaplan–Meier follow-up was 63 months (IQR = 47–89 months, 95% CI = 35.7–90.3 months). Primary tumors were from the genitourinary tract lesions including 46 (98%) lower tract SCCUT and one (2%) upper tract SCCUT.

The pathologic stage of SCCUT was pT4 in 12.8% (6/47), pT3 in 36.1% (17/47), pT2 in 36.1% (17/47), and pT1 in 6.4% (3/47); in four cases (8.5%), the final stage was not available (pTx). Pathologic lymph node assessment revealed N3 in 2% (1/47), N2 in 11% (5/47), N1 in 23% (11/47), and N0 in 64% (30/47). Distant metastasis was found in 17% (8/47), and 83% (39/47) were metastasis-free. Platinum-based chemotherapy was administered to 76.6% (36/47), 2.1% (1/47) received gemcitabine only, and 21.3% (10/47) did not receive chemotherapy. Radical cystectomy and nephroureterectomy were performed on 34% (16/47) and 2.1% (1/47) of patients, respectively; 64% (30/47) had transurethral resection of tumor (TUR) only.

Pure SCCUT was diagnosed in 38.3% (18/47) of cases, and the remaining 62% (29/47) revealed a mixture of SCCUT and conventional high-grade UC or other UC variants. The mixed SCCUT cases in this cohort contained between 30 and 90% of small cell component.

**Figure 1:**
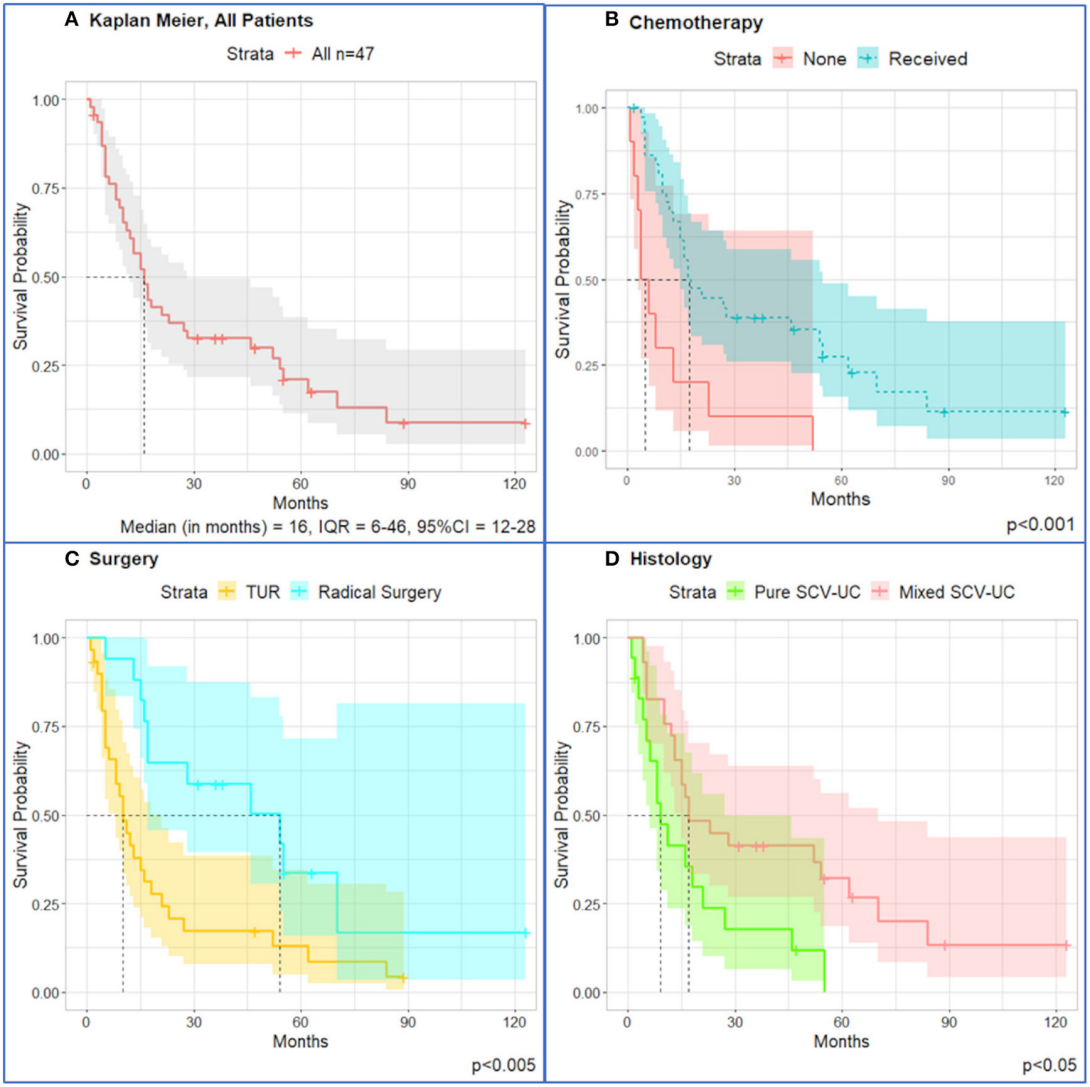
Kaplan–Meier survival curves for all patients (*n* = 47) diagnosed with SCCUT. **(A)** All patients, *n* = 47. **(B)** Patients who received chemotherapy lived longer (median = 17.5, IQR = 10–47) than those who did not undergo chemotherapy (median = 5.0, IQR = 3.3–11.8). **(C)** TUR only had worse outcomes (median = 10, IQR = 5–21) than radical surgery (median = 54, IQR = 17–70). **(D)** Diagnosis of pure SCCUT is a morbid survival (median = 9, IQR = 5–21) compared to mixed SCCUT (median = 17, IQR = 12–70). Shaded area is 95% confidence interval. Dotted lines represent the median overall survival.

Survival curves for all patients (*n* = 47) are shown in Figure 1. One-year survival is 59.6%, and median OS for the entire cohort is 16 months. Median OS of patients who received chemotherapy is 19.5 months (IQR = 12–62 months, 95% CI = 16–62 months), and patients who did not receive chemotherapy had median OS of 5.5 months (IQR = 3.5–10.5 months, 95% CI = 4 months to n/a) (*p* < 0.0001). Patients undergoing radical surgery had longer OS with median of 54 months (IQR = 17–70 months, 95% CI = 17 months to not reached) than patients undergoing TUR only, with median of 10 months (IQR = 5–21 months, 95% CI = 8–18 months) (*p* < 0.005). Patients diagnosed with mixed SCCUT had median OS of 17 months (IQR = 12–70 months, 95% CI = 15–70 months) and a better outcome than pure SCCUT with median OS of 9 months (IQR = 5–21 months, 95% CI = 6–27 months) (*p* < 0.05).

Univariate Cox proportional hazards analysis showed correlation between chemotherapeutic treatment [hazard ratio (HR) = 0.015, 95% CI = 0.0018–0.13, *p* < 0.001], treatment by radical surgery (HR = 0.37, 95% CI = 0.18–0.76, *p* < 0.01), and mixed SCCUT histology (HR = 0.44, 95% CI = 0.22–0.86, *p* = 0.02) with improved survival, whereas the presence of metastatic disease was associated with worse survival (HR = 2.1, 95% CI = 1.1–4.2, *p* = 0.03) (Table 2).

**Table 2:** Log-rank univariate analyses clinicopathologic features with overall survival for all patients diagnosed with SCCUT (*n* = 47) and patients with tissue available (*n* = 33).

	**All SCCUT (*****n*** **=** **47)**		**Tissue available (*****n*** **=** **33)**	
**Feature**	**HR (95% CI)**	***p*-value**	**HR (95% CI)**	***p*-value**
Age (y)				
≤70	Ref.		Ref.	
>70	1.5 (0.75–3)	0.25	2.6 (1.2–5.8)	0.02
Sex				
Male	Ref.		Ref.	
Female	1.3 (0.66–2.6)	0.43	0.93 (0.4 −2.1)	0.86
Chemotherapy			
None	Ref.		Ref.	
Received	0.29 (0.14–0.61)	0.001	0.015 (0.0018–0.13)	0.001
Surgery				
TUR only	Ref.		Ref.	
Radical	0.37 (0.18–0.76)	0.007	0.52 (0.24–1.2)	0.11
Recurrence				
No recurrence	Ref.		Ref.	
Recurrence	0.57 (0.29–1.1)	0.092	0.54 (0.25–1.2)	0.11
Stage				
≤pT1	Ref.		Ref.	
≥pT2	1.8 (0.7–4.6)	0.23	2 (0.67–5.7)	0.22
Nodes				
N0	Ref.		Ref.	
≥N1	1.6 (0.82–3.1)	0.17	1.2 (0.56–2.7)	0.61
Metastasis				
M0	Ref.		Ref.	
M1	2.1 (1.1–4.2)	0.03	2.2 (0.72–6.6)	0.17
Histology				
Pure SCCUT	Ref.		Ref.	
Mixed SCCUT	0.44 (0.22–0.86)	0.02	0.46 (0.21–1)	0.059
PD-L1—tumor cells	—	—		
0 <50%	—	—	Ref.	
≥50%	—	—	0.98 (0.97–1)	0.01
PD-L1—infiltrating immune cells	—	—		
0 <50%	—	—	Ref.	
≥50%	—	—	0.99 (0.98–1)	0.32
p53 expression	—	—		
0	—	—	Ref.	
1–2+	—	—	1.033 (0.37–21.97)	0.316
p63 extent	—	—		
0–1	—	—	Ref.	
2+	—	—	0.75 (0.5–1.1)	0.16
p63 intensity	—	—		
0–1	—	—	Ref.	
2+	—	—	0.87 (0.58–1.3)	0.46
GATA3 extent	—	—		
0–1	—	—	Ref.	
2+	—	—	1.1 (0.76–1.6)	0.61
GATA3 intensity	—	—		
0–1	—	—	Ref.	
2+	—	—	0.98 (0.73–1.3)	0.92

*p ≤ 0.05 is considered significant*.

*CI, confidence interval; HR, hazard ratio; SCCUT, small cell carcinoma of the urinary tract; TUR, transurethral resection*.

Metastatic disease was confirmed by imaging studies in 17% (8/47) of patients: four female and four male patients. Pure SCCUT was found in five of eight cases. One patient had pT4, three were pT3, and three were pT2, and in one patient, the degree of invasion could not be assessed (pTx). The most common metastatic sites included bone involvement in seven of eight, six of whom showed metastases to the thoracic and cervical spines, one patient showing involvement of the right hemipelvis. Two patients presented or progressed with liver and bone metastasis. One patient showed liver and lung metastasis and cervical adenopathy. Maximum survival in all metastatic patients was 23 months. The median OS for patients with metastatic disease (M1+) was 9 months (95% CI = 0–22.9 months, IQR = 5–16 months), and for patients with no signs of metastasis (M0), it was 17 months (95% CI = 8–26 months, IQR = 8–62 months) (*p* = 0.03).

### Immunohistochemistry

**Figure 2:**
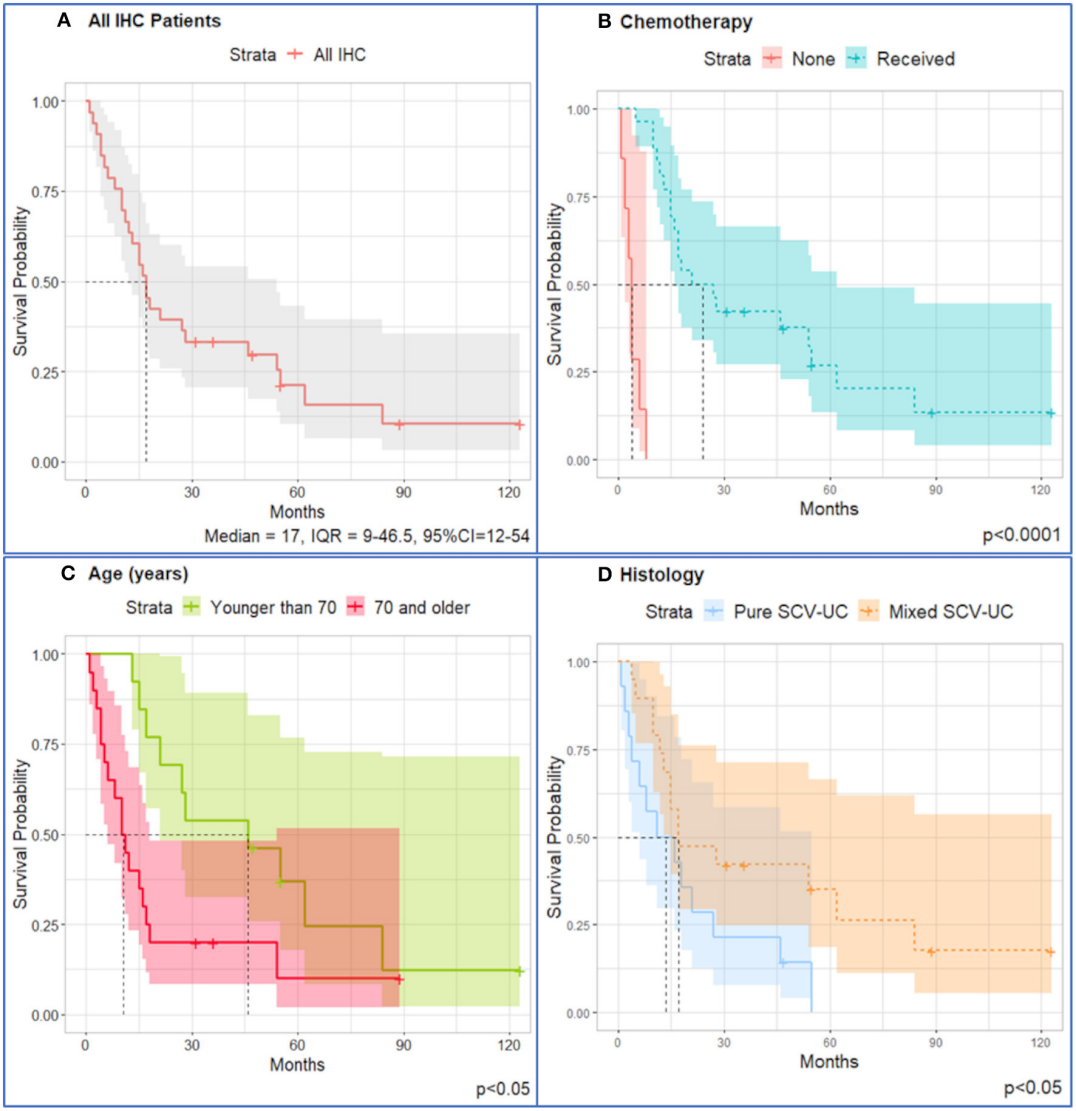
Kaplan–Meier survival curves for IHC patients (*n* = 33). **(A)** All patients; stratified by **(B)** chemotherapy (median = 24, IQR = 15–62) and no chemotherapy received (median = 4, IQR = 2–6); **(C)** patients younger than 70 years (median = 46, IQR = 21–62) have better survival than patients older than 70 years (median = 10.5, IQR = 4.5–17.5); **(D)** patients diagnosed with mixed SCCUT live longer (median overall survival = 17, IQR = 12–84) than patients diagnosed with pure SCCUT (median overall survival = 13.5, IQR = 4–27). Shaded area represents 95% confidence intervals. Dotted lines represent the median overall survival.

Tissue was available for 33 of 47 patients (70%) and immunohistochemical expression was evaluated. For patients with available tissue (*n* = 33), the median OS was 17 months (95% CI = 12–54 months, IQR = 9–46.5 months) (Figure 2).

Comparison of the means using analysis of variance (ANOVA) revealed that common clinicopathologic features are not different between patients with tissue available and the whole patient population. The pertinent clinicopathologic features are detailed in Table 1. The immunohistochemical profiles are summarized and compared in Table 3. All patents expressed high PD-L1 status: 97% (32/33) revealed ≥25% PD-L1 expression in TCs (most of them showing concomitant ICs+ ≥10%); one case revealed <1% expression in TCs and ICs+ in SCCUT; however, the non-small cell component in the same case revealed >25% PD-L1 expression in TCs; therefore, the case was considered, overall, PD-L1 high. Otherwise, among the non-pure SCCUT cases, the expressions of PD-L1 were comparable between small and non-small cell components.

**Table 3:** Immunohistochemical information (*n* = 33).

		**All IHC**	**Pure SCCUT**	**Mixed SCCUT**	***p*-value**
	N	33	14 (42.4%)	19 (57.6%)	
PD-L1—tumor cells	Median	70%	65.00%	75.00%	0.81
	Mean	64.60%	63.20%	65.50%	
	SD	27.90%	26.60%	28.40%	
	IQR	40–90%	37.5–90%	40–90%	
PD-L1—infiltrating immune cells	Median	50%	30.00%	70.00%	0.33
	Mean	49.70%	43.60%	54.20%	
	SD	31%	32.50%	29.10%	
	IQR	20–81%	10–82.5%	20–80%	
p53 expression	0	2 (6.1)	—	2 (10.5%)	0.22
	1	7 (21.2%)	5 (35.7%)	2 (10.5%)	
	2	24 (72.7%)	9 (64.3%)	15 (78.9%)	
p63—intensity	0	14 (42.4%)	9 (64.3%)	5 (26.3%)	0.04
	1	12 (36.4%)	4 (28.6%)	8 (42.1%)	
	2	3 (9.1%)	—	3 (15.8)	
	3	4 (12.1%)	1 (7.1%)	3 (15.8)	
p63—extent	0	14 (42.4%)	9 (64.3%)	5 (26.3%)	0.03
	1	10 (30.3%)	3 (21.4%)	7 (36.8%)	
	2	7 (21.2%)	2 (14.3%)	5 (26.3%)	
	3	2 (6.1%)	—	2 (10.5%)	
GATA3—intensity	0	18 (54.5%)	10 (71.4%)	8 (42.1%)	0.16
	1	4 (12.1%)	1 (7.1%)	3 (15.8%)	
	2	4 (12.1%)	1 (7.1%)	3 (15.8%)	
	3	7 (21.2%)	2 (14.3%)	5 (26.3%)	
GATA3—extent	0	18 (54.5%)	10 (71.4%)	8 (42.1%)	0.18
	1	8 (24.2%)	2 (14.3%)	5 (26.3%)	
	2	3 (9.1%)	1 (7.1%)	2 (10.5%)	
	3	4 (12.1%)	1 (7.1%)	3 (15.8%)	

*Stratified by histological subtype. Expression of p63 (0.03) is different between the two histologies. Median, mean, frequency, percentage, standard deviation, and IQR are listed where applicable. p ≤ 0.05 is considered significant*.

*IHC, immunohistochemistry; SCCUT, small cell carcinoma of the urinary tract*.

**Figure 3:**
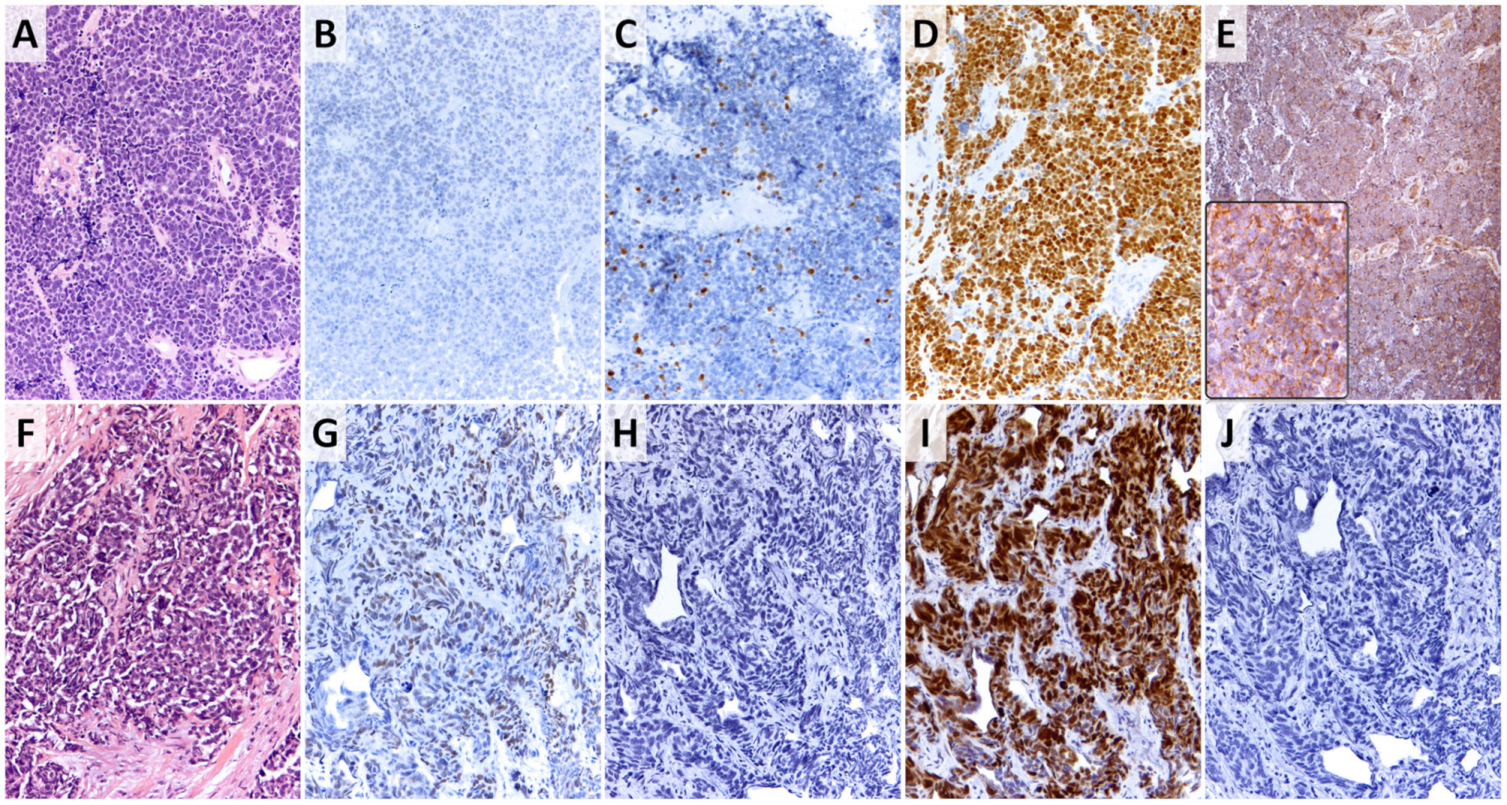
Small cell variant of urothelial carcinoma. **(A,F)** Tumor morphologies (hematoxylin-eosin stain, 200×); **(B,G)** p63 expression as the surrogate marker for basal phenotype in SCCUT (panel B shows no expression and panel G shows focal moderate expression) (p63 stain, 200×); **(C,H)** GATA3 expression as the surrogate marker for luminal phenotype in SCCUT (panel C shows focal strong expression and panel H shows absence of expression) (GATA3 stain, 200×); **(D,I)** p53 expression in SCCUT [both panels show increased (2+) expression] (p53 stain, 200×); **(E,J)** PDL1 expression in SCCUT (panel E and inset show 90% expression in tumor cells and >50% expression in immune cells, and panel J shows absence of expression in tumor cells and infiltrating immune cells) (PDL1 stain, 200×, inset = 400×).

Figure 3 shows representative images of GATA3, p63, p53, and PD-L1 expressions.

Among the cases, 72.7% (24/33) revealed overexpression of p53 (score 2+), 21.2% (7/33) had wild-type (score 1+) p53 expression, and total loss of p53 (score 0) was noted in 6.1% (2/33).

Expression of p53 (2+) was noticed concurrently in 15.2% (5/33) with GATA3 surrogate for luminal phenotype, 27.3% (9/33) with p63 surrogate for basilar phenotype, and in 15.2% (5/33) with both GATA3 and p63 overexpression. The remaining 15.2% (5/33) revealed p53 (2+) expression with no expression of GATA3 or p63. Wild-type p53 expression (score 1+) was concomitantly present with p63 and GATA3 in 6.1% (2/33), with p63 expression alone in 3% (1/33), and with GATA3 alone in 3% (1/33). Loss of expression of p53 was seen in 6.1% (2/33) of patient samples expressing both p63 and GATA3. There was no meaningful difference between expressions of p53 in basal or luminal tumors.

Univariate Cox proportional hazards analysis (Table 2) revealed significant protective effects for chemotherapy (HR = 0.015, 95% CI = 0.0018–0.13, *p* < 0.001) and elevated PD-L1 expression in TCs (HR = 0.98, 95% CI = 0.97–1.00, *p* = 0.01). Age older than 70 years was an adverse risk (HR = 2.6, 95% CI = 1.2–5.8, *p* = 0.02). A diagnosis of mixed SCCUT has some protective effects on survival (HR = 0.21, 95% CI = 0.21–1.0, *p* = 0.059). There were no significant associations between p53, GATA3, or p63 and survival.

## Discussion

SCCUT is a rare variant of UC with aggressive clinical course and poor prognosis. To our knowledge, this is the first study to evaluate PD-L1 expression specifically in SCCUT and present an immunohistochemical classification based on molecular subtype. The examined SCCUT tumors were characterized predominantly (97%) by a high PD-L1 phenotype regardless of histology, treatment history, or p53 status. Univariate analysis identified increased hazards for older patients (>70 years old) and metastatic disease. Improved OS was exhibited from chemotherapy, radical surgery, diagnosis of mixed SCCUT, and high PD-L1 expression in TCs.

As an immune escape mechanism, it is counterintuitive that high PD-L1 expression in TCs improves survival. Thus, given the overall risk reduction was 2% and the CI (0.97–1.00), it is likely that the effect is not clinically significant. Moreover, these findings do not align with the available literature. A large meta-analysis by Zhao et al. including 3,674 patients from 18 studies found that PD-L1 expression in ICs+ and not TCs was, indeed, protective and imparted positive prognostic value (10). Further clinical investigations at other institutions comparing pure and mixed SCCUT will shed light on the association of PD-L1 expression in TCs with OS.

Contrary to our study, the study by Pichler et al. on 48 pure UC and 13 variants suggested that elevated PD-L1 expression was associated with diminished chemotherapy response (11). However, elevated PD-L1, more commonly found in advanced, aggressive bladder carcinomas, suggests a correlation between PD-L1 expression and tumor grade and stage (12, 13). Unlike our finding of prominent PD-L1–high phenotype in SCCUT patients, the study by Mandelkow et al. on 12 samples of SCCUT did not identify any expression of PD-L1 (14). Little is known about the patient demographics, clinicopathologic features, and archival specimen that were retrieved in their study.

There are data from Yang et al. suggesting that targeting the adenosine receptor A2A (ADORA2A), involved in cytotoxic TCD8 anergy, may be a rational drug development approach for SCCUT, as this receptor was expressed in 34 samples of SCCUT (15). These 34 samples showed no IC infiltration, qualifying as an “immune desert.” Yang et al. also reported that SCCUT revealed downregulation of both basal and luminal markers, therefore referred to as double negative (15). The authors argue that this may represent a urothelial-to-neural phenotype switch. One would argue that the change of phenotype to neural should result in higher expression of neuroendocrine markers in the SCCUT that is not often present (16). In addition, we identified frequent expression of basal and luminal markers in SCCUT (p63 and GATA3, respectively). The differences in findings might be due to the heterogeneous nature of bladder carcinomas, the difference in the antibodies used, technique of staining and interpretation of stain, and other individual patient variables that were not considered, such as genetic profile. Almost all of the tested specimens in our study expressed a PD-L1–high phenotype suggesting that SCCUT patients can benefit from anti-PD-L1 immunotherapy. In the only case with absence of PD-L1 expression in the SCCUT, the non-small cell component revealed a PD-L1–high phenotype, making the patient eligible for immunotherapy.

Anti-PD-L1 immunotherapy with PD-1/PD-L1 inhibitors is approved for treatment of UC in cisplatin ineligible patients regardless of PD-L1 expression (3). The IMvigor 210 trial included 119 patients receiving atezolizumab as first-line treatment for advanced or metastatic UC and demonstrated an objective response rate of 23% and included 9% of patients with complete response (17). The seminal reference, however, continues to be Keynote-045, which compared pembrolizumab with physician option of chemotherapy showing greatly improved survival in the patients receiving immunotherapy (18, 19). Data from IMVigor010 presented at the 2020 Annual Meeting of the Genitourinary section of the American Society of Clinical Oncology showed no benefit with adjuvant atezolizumab after neoadjuvant cisplatin-based chemotherapy followed by radical cystectomy (20). However, we wait for data with adjuvant nivolumab in the same setting from CheckMate-274 and the phase III AMBASSADOR trial, which investigates adjuvant pembrolizumab after radical cystectomy in patients with localized high-risk muscle-invasive bladder cancer or upper tract UC (21, 22). Clinical trials continue to demonstrate the utility of immune checkpoint blockade as a treatment strategy in UC, although no study has focused on SCCUT.

Plimack et al. included a small cohort of UC variants in their study (three cases, 9% of the cohort, one showing SCCUT) and report significant antitumor activity and acceptable safety for pembrolizumab in advanced UC and its variants (23). The authors did not discuss the fate of the variants in their study. Another single case report has reported striking clinical response to pembrolizumab in a patient with treatment-refractory SCCUT (24).

The median OS in SCCUT has been reported in a range from 5.3 to 34.9 months with a 5-year survival rate ranging from 0 to 40%. (2, 25–29). Bhatt et al. identified a median OS of 11 months and 5-year survival of 0% in a cohort of 14 patients with SCCUT; Wang et al. reported median OS of 29 months in a cohort of 81 patients, and Choong et al. reported median OS of 20 months in a cohort of 44 patients. In a cohort of 960 patients selected from the National Cancer Database, Geynisman et al. report median OS of 8.6 months (30). Our study reports a median OS of 17 months with a 1-year survival rate of 59.6% in a cohort of 47 patients.

It is not clear why a non-small cell component mixed to SCCUT is a good prognostic factor. In patients with tissue available, the differences in clinicopathologic features between the two major histological groups—SSCUT and mixed SSCUT—are summarized in Table 4. Considering chemotherapy, which provided a 98% risk reduction (Table 2), six patients with pure SCCUT did not receive chemotherapy, whereas only one patient with mixed SCCUT did not (*p* = 0.011). All chemotherapy administered was platinum based and administered in combination with at least one other agent in every patient (Table 4). Furthermore, mixed SCCUT patients underwent radical surgery at greater rate than pure SCCUT (58 vs. 21%, *p* = 0.04). Overall, the median survival for SSCUT and mixed SSCUT were 13.5 and 17 months, respectively (*p* = 0.1). These diverse observations indicate that as a whole, chemotherapy was protective; however, no direct comparison can be made with surgery.

**Table 4:** Clinicopathologic features of patients with tissue (*n* = 33), stratified by histology.

**Patients with immunohistochemistry**		**Pure SCV-UC**	**Mixed SCV-UC**	***p-*value**
No. of Patients		14 (100%)	19 (100%)	−
Survival (months)	Median	13.5	17	0.1
	Mean	18.93	35.8	
	SD	18.3	33.5	
	IQR	4.5, 25.5	12.5, 54.5	
Age (year)	Median	74	71	0.94
	Mean	72.3	72.1	
	SD	9.6	9.1	
	IQR	64.3, 79.3	64.5, 79.5	
Sex	Female	7 (50%)	3 (16%)	0.04
	Male	7 (50%)	16 (84%)	
Chemotherapy	None	6 (43%)	1 (5.3%)	0.01
	Etoposide and carboplatin	5 (36%	8 (42.1%)	
	MVAC	1 (7%)	2 (10.6%)	
	Etoposide, carboplatin, and cisplatin	1 (7%)	0	
	Gemcitabine and cisplatin	1 (7%)	3 (16%)	
	Carboplatin and paclitaxel	0	2 (10.6%)	
	Etoposide and cisplatin	0	3 (16%)	
Surgery	TURBT	11 (79%)	8 (42.1%)	0.04
	RC	3 (21%)	10 (52.7%)	
	Nephro-U	0	1 (5.3%)	
Primary site	Bladder	14 (100%)	18 (94.7%)	0.4
	Ureter	0	1 (5.3%)	
History of recurrence	No recurrence	6 (43%)	9 (47.4%)	0.81
	Recurrence	8 (57%)	10 (52.6%)	
Pathologic stage	Tx	4 (29%)	0	0.096
	T1	1 (7%)	2 (10.6%)	
	T2	2 (14%)	4 (21.2%)	
	T3	4 (29%)	11 (57.9%)	
	T4	3 (21%)	2 (10.6%)	
Nodes	N0	11 (79%)	10 (52.5%)	0.08
	N1	3 (21%)	6 (31.5%)	
	N2	0	3 (16%)	
Metastasis	M0	11 (79%)	18 (94.7%)	0.17
	M1	3 (21%)	1 (5.3%)	

*Mean, standard deviation, median, IQR, frequency, and percent are listed where applicable. Pathological TNM staging information is based on the guidelines of the AJCC 7th edition Cancer Staging Manual. p ≤ 0.05 is considered significant*.

*SCV, small cell variant; UC, urothelial carcinoma; TUR = transurethral resection; RC, radical cystectomy; Nephro-U, nephroureterectomy*.

The three subtypes explored in this study include p53 wild-type, basal-like, and luminal-like. GATA3, a marker of luminal type, is correlated with better outcomes compared to basal-like and p53 wild-type tumors (7). Expression of p63 protein, typical in basal subtypes, is correlated with higher tumor stage and aggressive behavior and has been described as a negative predictive factor in patients diagnosed with UC (8, 31, 32). In our cohort, both p63 and GATA3 were detected at greater frequency in the mixed SCCUT patients than in pure SCCUT. Therefore, these data are inconclusive in grouping SCCUT into luminal or basal subtypes.

Two patients with mixed SCCUT did not express any p53, demonstrating a truncation or deletion of the gene. However, this was not a statistically significant finding. Overexpression of protein p53 was found in 64.3% of pure SCCUT and in 78.9% of mixed SCCUT patients, suggesting that an abnormal or upregulated p53 transcription is present in these patients. These findings are most consistent with the p53-like subtypes. Overexpression has been correlated with chemotherapy resistance, and intact non-mutated expression is associated with worse survival than a mutated type (31, 33).

As there are more than 12 isoforms of p53 with distinct functions (i.e., Δ133p53α impairs senescence and enhances angiogenesis; p53β induces apoptosis), analysis of p53 expression and the specific mutation might help identify patients who are likely to develop metastatic SCCUT (34). It may be that there is a positive relation between Δ133p53α expression and metastatic disease; similarly, a longer survival might be associated with elevated p53β in patients diagnosed with SCCUT. Specific isoforms of p53 have yet to be tested for in SCCUT, but studies of other cancers have found prognostic value in the unique patterns of expression (34).

A recent publication by Kadosh et al. shows that mutant p53 constitutively suppresses the oncogenic Wnt/β-catenin pathway in the upper gastrointestinal tract; in the lower intestinal tract, however, bacteria carrying an enzyme named shikimate produce gallic acid, which regulates the Wnt gene promoter and nullifies the p53 effect on growth suppression, likely potentiating additional carcinogenic pathways (33). This production of gallic acid mimics the action of Δ133p53α and Δ40p53α (34). It is thought provoking to see if environmental exposures or external events such as the microbiome could similarly modulate mutant p53 in SCCUT, especially by recognizing gene signatures linked to exposures in the small cell variant of the UC genomes (35).

The herein presented study is limited as it deals with a rare disease. Actually, the low incidence of SCCUT begs for a retrospective study investigating all historical accounts of this malignancy in order to improve guidance in prospective cases. However, the low incidence of SCCUT and the nature of a retrospective study imply that several valuable clinical questions remain unanswered. Accurate comparisons of treatment cannot be made from this cohort as there were new developments made during the 29-year period included in the search, including improved treatment modalities. Thus, matched and balanced comparisons as befit a dynamically randomized clinical trial are near impossible in retrospective studies, especially when the sample size is small. In this study, the small sample size influences the reliability of additional analyses including detailed comparisons of patients with recurrence, metastasis, and chemotherapy type. Retrospective studies combining information from multiple institutions can generate a dataset large enough to withstand balancing and more clinically significant analyses including stage and treatment-matched cancer-specific survival.

One of the greatest pitfalls of a retrospective study, especially one that investigates patients prior to the current information age, before the adoption of electronic record keeping, is the inconsistencies in record keeping. These concerns are apparent in this study as 30% of patients did not have adequate specimens for immunohistochemical processing. Knowing these limitations, as we continue to explore new treatment modalities and improve outcomes, it is of utmost importance that record keeping continues to improve. The final limitation in this study was in antibody selection as there are several clones available; however, neither all are commercially available nor used in clinical practice. It would be interesting to see expressions of additional clones of antibodies targeting p63, GATA3, p53, or PD-L1 in future studies of SCCUT.

Our group aims to circumvent some of these shortcomings by describing gene signatures in SCCUT, compare its gene expression profile to demographically and clinically matched patients with small cell carcinoma of the lung, as well as patients with UC, and continue to enroll advanced SCCUT into clinical trials with an immune checkpoint backbone.

## Conclusions

To our knowledge, this is the first cohort of SCCUT demonstrating high expression of PD-L1. Approximately two-thirds of SCCUT in our series revealed a phenotype of p53 overexpression, which would potentially result in poor response to chemotherapy; therefore, the finding of remarkably high PD-L1 phenotypes in this cohort highlights important clinical implications, suggesting that SCCUT may be targetable by anti-PD-L1 therapies. Further study of SCCUT will provide insight into prognostic markers and therapeutic targets.

## Data Availability Statement

The raw data supporting the conclusions of this article will be made available by the authors, without undue reservation.

## Ethics Statement

The studies involving human participants were reviewed and approved by The Miriam Hospital Institutional Review Board. Written informed consent for participation was not required for this study in accordance with the national legislation and the institutional requirements.

## Author Contributions

BGo and AA conceptualized the study. All authors aided in data collection, review, and analysis. All authors wrote and reviewed the manuscript.

## Conflict of Interest

The authors declare that the research was conducted in the absence of any commercial or financial relationships that could be construed as a potential conflict of interest.
